# Fli1 Represses Transcription of the Human α2(I) Collagen Gene by Recruitment of the HDAC1/p300 Complex

**DOI:** 10.1371/journal.pone.0074930

**Published:** 2013-09-13

**Authors:** Yoshihide Asano, Maria Trojanowska

**Affiliations:** 1 Department of Dermatology, University of Tokyo Graduate School of Medicine, Tokyo, Japan; 2 Arthritis Center, Boston University School of Medicine, Boston, Massachusetts, United States of America; University of Insubria, Italy

## Abstract

Fli1, a member of the Ets transcription factor family, is a key repressor of the human α2(I) collagen (COL1A2) gene. Although our previous studies have delineated that TGF-β induces displacement of Fli1 from the COL1A2 promoter through sequential post-translational modifications, the detailed mechanism by which Fli1 functions as a potent transcriptional repressor of the COL1A2 gene has not been fully investigated. To address this issue, we carried out a series of experiments especially focusing on protein-protein interaction and epigenetic transcriptional regulation. The combination of tandem affinity purification and mass spectrometry identified HDAC1 as a Fli1 interacting protein. Under quiescent conditions, HDAC1 induced deacetylation of Fli1 resulting in an increase of Fli1 DNA binding ability and p300 enhanced this process by promoting the formation of a Fli1-HDAC1-p300 complex. TGF-β-induced phosphorylation of Fli1 at threonine 312 led to disassembly of this protein complex. In quiescent dermal fibroblasts Fli1, HDAC1, and p300 occupied the −404 to −237 region, including the Fli1 binding site, of the COL1A2 promoter. TGF-β induced Fli1 and HDAC1 dissociation from the COL1A2 promoter, while promoting Ets1 and p300 recruitment. Furthermore, acetylation levels of histone H3 around the Fli1 binding site in the COL1A2 promoter inversely correlated with the DNA occupancy of Fli1 and HDAC1, while positively correlating with that of Ets1 and p300. In the functional studies, HDAC1 overexpression magnified the inhibitory effect of Fli1 on the COL1A2 promoter. Moreover, pharmacological blockade of HDAC1 by entinostat enhanced collagen production in dermal fibroblasts. Collectively, these results indicate that under quiescent conditions Fli1 recruits HDAC1/p300 to the COL1A2 promoter and suppresses the expression of the COL1A2 gene by chromatin remodeling through histone deacetylation. TGF-β-dependent phosphorylation of Fli1 at threonine 312 is a critical step regulating the remodeling of the Fli1 transcription repressor complex, leading to transcriptional activation of the COL1A2 gene.

## Introduction

Fli1 is a member of the Ets transcription factor family that was initially identified as a proto-oncogene in Friend virus-induced erythroleukemia in mice [1], [2]. In humans, Fli1 is frequently involved in the development of Ewing sarcoma and related subtypes of primitive neuroectodermal tumors [3], suggesting that Fli1 plays an important role in the process of cellular transformation. In normal tissues, Fli1 is preferentially expressed in endothelial and hematopoietic cell lineages [4] and participates in the regulation of the development and differentiation of these cell types. Extensive *in vitro* studies as well as the data obtained from various Fli1 mutant mice support a crucial role of Fli1 in megakaryocytic differentiation and myelomonocytic, erythroid, and NK cell development [5]–[8]. Furthermore, Fli1 knockout mice die during embryogenesis with a loss of vascular integrity leading to cerebral hemorrhage, suggesting that Fli1 is involved in the regulation of genes critical for vascular remodeling [5]. Endothelial Fli1 deficiency affects vascular homeostasis through the impairment of endothelial adhesion junctions, vascular basement membrane remodeling, and endothelial cell-pericyte interactions as described in our previous report [9].

Although Fli1 is present in a relatively limited amount in dermal fibroblasts, Fli1 plays a pivotal role in the regulation of the extracellular matrix (ECM) and its related molecules, including type I collagen [10]–[12], tenascin-C [13], [14], ECM-degrading enzymes [15]–[17] and the multifunctional matricellular factor CCN2 [18]. Most importantly, Fli1 has been shown to be a potent inhibitor of type I collagen production in dermal fibroblasts and persistent downregulation of this transcription factor has been implicated in the pathogenesis of cutaneous fibrosis in scleroderma [12], [19]. The data from our laboratory and others demonstrated that Fli1 binds to the human α2(I) collagen (COL1A2) promoter under physiological conditions, but dissociates from the promoter in response to TGF-β stimulation [10], [11]. Given that gene silencing of Fli1 results in a robust increase of type I collagen production [18], this process is critical for the TGF-β-dependent up-regulation of the type I collagen gene. Our recent studies have elucidated the principal mechanism responsible for TGF-β-dependent regulation of Fli1 activity in the context of the COL1A2 promoter [20]–[22]. This pathway is initiated by TGF-β-dependent activation of c-Abl tyrosine kinase that mediates nuclear translocation of protein kinase C-δ (PKC-δ) and phosphorylation of Fli1 at threonine 312. This modification triggers subsequent Fli1 acetylation at lysine 380 by p300/CREB-binding protein-associated factor (PCAF) and displacement of Fli1 from the COL1A2 promoter. However, the mechanism through which Fli1 exerts its potent inhibitory effect on COL1A2 gene expression has not yet been elucidated.

Earlier studies from our laboratory and others have suggested that Fli1 and Ets1 compete for the binding of the Ets binding domain in the COL1A2 promoter and the ratio of Ets1/Fli1 plays an important role in the regulation of promoter activity [10]. However, our earlier study also demonstrated that the Fli1 W321R mutant, which does not bind to DNA, as well as a deletion construct containing only the Ets domain exert a more modest but consistently reproducible inhibitory effect on COL1A2 promoter activity compared with wild type Fli1, suggesting that an indirect mechanism as well as a direct mechanism contribute to the inhibitory effects of Fli1 on the COL1A2 promoter activity. The purpose of this study was to further investigate the molecular mechanism by which Fli1 exerts its inhibitory effect on COL1A2 gene. By a series of experiments, we demonstrated that Fli1 recruits HDAC1 to the COL1A2 promoter and decreases the acetylation levels of histone H3. These results indicate that Fli1 functions as a potent transcriptional repressor through chromatin modification as well as competition with transcriptional activator Ets1.

## Materials and Methods

### Ethics Statement

Human dermal fibroblast cultures were established from the foreskins of healthy newborns from the Medical University of South Carolina Hospital. This study was approved by the Medical University of South Carolina Institutional Review Board for Human Research, and written informed consent was obtained from both parents of the child. The whole study was performed according to the Declaration of Helsinki.

### Reagents

Recombinant human TGF-β1 was obtained from PeproTech (Rocky Hill, NJ, USA). The polyclonal rabbit anti-Fli1 antibody was described previously [8]. The monoclonal mouse anti-Fli1 antibody was purchased from BD Bioscience (Bedford, MA, USA). The polyclonal rabbit anti-acetylated lysine antibodies, anti-HA tag antibody and anti-HDAC1 antibody were purchased from Cell Signaling Technology (Danvers, MA, USA). Antibodies for β-actin and Flag tag were purchased from Sigma-Aldrich (St. Louis, MO, USA). Antibodies for p300, PCAF, and Ets1 were obtained from Santa Cruz Biotechnology (Santa Cruz, CA, USA). The antibody for calmodulin binding peptide was purchased from Millipore (Billerica, MA, USA). Entinostat was purchased from Selleck Chemicals (Houston, TX, USA). Adenoviral vectors expressing Fli1, green fluorescence protein, Fli1 siRNA or scrambled non-silencing RNA were generated as described previously [12], [23].

### Cell Cultures

Human dermal fibroblast cultures were established from the foreskins of healthy newborns from the Medical University of South Carolina Hospital as described previously [21]. All studies used cells from passage number 3 to 6. Before stimulation with TGF-β or entinostat, fibroblasts were incubated in serum-free medium for 48 hours. Human embryonic kidney 293T cells were purchased from ATCC and maintained in DMEM supplemented with 10% fetal bovine serum.

### Plasmid Construction

pSG5-Fli1 and pCTAP-Fli1, which has two different tags, including a streptavidin binding peptide (SBP) and a calmodulin binding peptide, were described previously [20]. A -772 COL1A2/CAT construct was generated as previously described [5]. The expression vector of p300 was a gift from Dr Boyes (Institute of Cancer Research, London, UK). Expression vectors of PCAF and PCAF/ΔHAT, which lacks histone acetyltransferase (HAT) activity, were gifts from Dr Kouzarides (Cambridge University, Cambridge, UK). Plasmids were purified twice on cesium chloride gradients. At least two different plasmid preparations were used for each experiment.

### Protein Isolation by Tandem Affinity Purification and Mass Spectrometry

The InterPlay Mammalian TAP System (Stratagene, La Jolla, CA, USA) was employed according to the manufacturer’s instructions. The cDNA encoding human the Fli1 gene and two tandem affinity tags derived from the pCTAP-Fli1 vector was cloned into adenoviral shuttle vectors and linearized for *in vitro* ligation with the adenoviral backbone construct lacking the E1, E3, and E4 regions of the adenovirus genome. The pCTAP-Fli1 adenovirus expresses green fluorescence protein and tagged Fli1 under the control of two separate cytomegalovirus promoter/enhancers. Human dermal fibroblasts (1×10^8^) were transduced with the pCTAP-Fli1 adenovirus. After 72 hours, cells were harvested and resuspended in lysis buffer supplemented with protease inhibitors. Cells underwent three rounds of freeze-thawing, cell debris was pelleted by centrifugation at 16,000×g for 10 minutes, and the supernatant was collected. Next, EDTA (2 mM final concentration), β-mercaptoethanol (10 mM final concentration), and streptavidin resin were added to the cell lysate and incubated at 4°C for 2 hours. The resin was collected by centrifugation at 1,500×g for 5 minutes, washed twice, and incubated in streptavidin elution buffer for 30 minutes at 4°C. To further purify the protein complexes, calmodulin resin and calmodulin binding buffer were added to the supernatant. After 2-hour incubation at 4°C, the resin was collected (centrifugation at 1,500×g for 5 minutes) and washed twice. Then, protein complexes were eluted by adding calmodulin elution buffer and incubation for 30 minutes at 4°C. The resulting eluate was concentrated by trichloroacetic precipitation, resolved by two-dimensional gel electrophoresis, and visualized on the gel by silver staining. In a control experiment, the pull-down approach was performed with beads only. Bands which appeared on the gel from the Fli1 overexpression experiment but not the control experiment were excised and sent for mass spectrometry analysis to the Mass Spectrometry Facility at Medical University of South Carolina. According to the quality requirements of the facility, a protein can only be considered as an interactor if two or more peptides match the respective protein in the Swiss-Prot database.

### Immunoblotting

Whole cell extracts were prepared using lysis buffer with the following contents: 1% Triton X-100, 50 mM Tris-HCl [pH 7.4], 150 mM NaCl, 3 mM MgCl_2_, 1 mM CaCl_2_, proteinase inhibitor cocktail (Roche Applied Sciences, Indianapolis, IN, USA), 1 mM phenylmethyl sulfonyl fluoride and 100 ng/ml of Trichostatin A (Sigma-Aldrich). Protein extracts were subjected to SDS-PAGE and transferred to nitrocellulose membranes. Membranes were incubated overnight with primary antibody, washed, and incubated for 1 hour with secondary antibody. After washing, visualization was performed by enhanced chemiluminescence (Pierce, Rockford, IL, USA).

### Immunoprecipitation

To precipitate non-tagged proteins, whole cell extracts (500 µg) were pre-adsorbed with protein G sepharose beads (GE Healthcare, Waukesha, WI, USA) and incubated with 2 µg of appropriate antibodies and then with protein G sepharose beads. Streptavidin-coupled agarose beads (Sigma-Aldrich) were used instead of protein G sepharose beads for immunoprecipitation of ectopically expressed tagged-Fli1. The precipitated proteins were subjected to immunoblotting.

### *In vivo* Acetylation Assay

293 T cells were transfected with expression vectors encoding tagged-Fli1 (0.1 µg) and the indicated HAT protein (2 µg), and incubated for 48 hours. Whole cell lysates (500 µg) were incubated with 10 µl of streptavidin-coupled agarose beads overnight at 4°C. Precipitated proteins were subjected to immunoblotting using a rabbit anti-acetylated lysine antibody. After development, the membrane was stripped and reprobed with an anti-calmodulin binding peptide antibody to determine the total levels of ectopically expressed Fli1.

### DNA Affinity Precipitation Assay

DNA affinity precipitation (DNAP) was carried out as described previously [21]. Briefly, whole cell extracts (500 µg) prepared from 293 T cells were incubated for 10 minutes at 4°C with gel shift binding buffer (10 mM Tris-HCl [pH 8.0], 40 mM KCl, 1 mM DTT, 6% glycerol, 0.05% NP-40), and 20 µg of poly(dI-dC) (Pierce) in a final volume of 1 ml. Preclearing was performed by adding streptavidin-coupled agarose beads and incubating the mixture for 30 minutes with gentle rocking at 4°C. After centrifugation, the supernatant was incubated with 500 pmol of the COL1A2 Ets binding site (EBS) oligonucleotide overnight at 4°C with gentle rocking. Then, 65 µl of streptavidin-coated agarose beads was added, followed by a further 2-hour incubation at 4°C. The protein-DNA-streptavidin-agarose complex was washed twice with Tris-EDTA (100 mM NaCl), twice with gel shift binding buffer, and once with phosphate-buffered saline. The precipitates were subjected to immunoblotting using the anti-Fli1 antibody. The specific binding of Fli1 to the COL1A2 EBS oligonucleotide through EBS was demonstrated previously [11], [20].

### Chromatin Immunoprecipitation Assay

The chromatin immunoprecipitation (ChIP) assay was carried out essentially as described previously [18]. Briefly, cells were treated with 1% formaldehyde for 10 minutes. The cross-linked chromatin was then prepared and sonicated to an average size of 300–500 bp. The DNA fragments were immunoprecipitated overnight with or without polyclonal anti-Fli1 antibody at 4°C. After reversal of cross-linking, the immunoprecipitated chromatin was amplified by PCR amplification of specific regions of the COL1A2 genomic locus. The primers were as follows: COL1A2/F-404∶5′-CTGGACAGCTCCTGCTTTGAT-3′, COL1A2/R-233; 5′-CTTTCAAGGGGAAACTCTGACTC-3′. The amplified DNA products were resolved by agarose gel electrophoresis.

### Reverse Transcription Quantitative Real-time PCR

Total RNA was isolated from confluent fibroblasts using Tri reagent (MRC Inc., Cincinnati, OH, USA) according to the manufacturer’s instructions. Two µg of RNA was used for reverse transcription cDNA synthesis in a 20 µl reaction volume using random primers and then diluted to 40 µl. Quantitative real-time PCR was carried out using IQ Sybr green mix and an Icycler machine (BIO-RAD, Hercules, CA, USA) using 1 µl of diluted cDNA in triplicate with β-actin as the internal control. PCR conditions were 95°C for 3 minutes, followed by 40 cycles of 95°C for 30 seconds, 58°C for 1 minute. Melt curve analysis of the PCR products confirmed the absence of secondary products. The sequences of α1(I) collagen and β-actin primers were previously reported [18].

### Statistical Analysis

Data presented as bar graphs are the means ± SD of at least three independent experiments. Statistical analysis was performed using the Mann Whitney-U test (p<0.05 was considered significant).

## Results

### HDAC1 Interacts with Fli1 and Mediates its Deacetylation

**Figure 1:**
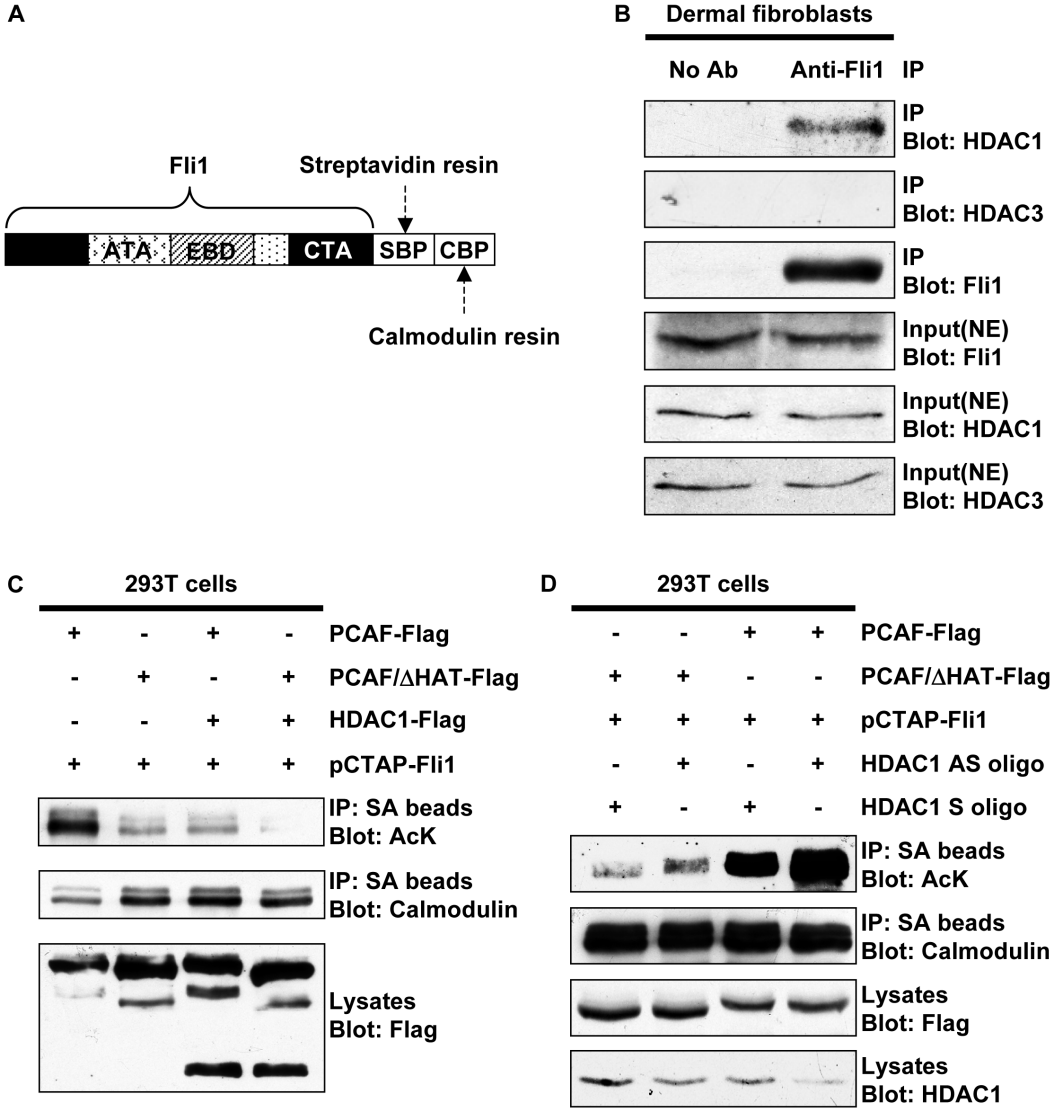
HDAC1 interacts with Fli1 and mediates its deacetylation. A. A schematic representation of pCTAP-Fli1. Streptavidin and calmodulin resins bind to streptavidin-binding peptide (SBP) and calmodulin-binding peptide (CBP), respectively. ATA: A-terminal activation domain, EBD: Ets binding domain, CTA: C-terminal activation domain. **B.** Foreskin fibroblasts were grown to subconfluence and serum-starved for 48 hours. Equal amount of protein from whole cell lysates was subjected to immunoprecipitation with or without anti-Fli1 antibody. The levels of Fli1, HDAC1, and HDAC3 proteins in precipitates were determined by immunoblotting. The levels of Fli1, HDAC1 and HDAC3 in whole cell lysates were determined by Western blotting. **C.** 293 T cells were transfected with pCTAP expression vector encoding wild type Fli1 along with PCAF, PCAFΔHAT, or HDAC1 expression vectors, and incubated for 48 hours. Total cell extracts were subjected to immunoprecipitation using streptavidin-coupled agarose beads (SA beads), followed by immunoblotting using rabbit anti-acetylated lysine antibody (AcK). To visualize the total levels of ectopically expressed Fli1, the same membrane was stripped and reprobed with anti-calmodulin binding peptide antibody. The levels of Flag-tagged proteins in cell lysates were determined by Western blotting. **D.** 293 T cells were transfected with pCTAP wild type Fli1 along with PCAF or PCAFΔHAT expression vectors. Six hours after the transfection, HDAC1 antisense oligonucleotide (HDAC1 AS oligo) or HDAC1 sense oligonucleotide (HDAC1 S oligo) were added to the cells. Whole cells lysates were prepared 48 hours after transfection. The levels of acetylated Fli1 and Flag-tagged proteins were determined as described above. The efficacy of HDAC1 AS oligo was evaluated by Western blotting.

As an initial experiment, we employed a proteomic analysis to identify nuclear factors that form a complex with Fli1 in dermal fibroblasts. To this end, adenovirus expressing Fli1 with two tags (Figure 1A) was transduced into dermal fibroblasts and the transcription factor complex including Fli1 was purified according to the manufacturer’s instructions. Purified protein complex was subjected to two-dimensional gel electrophoresis and several spots were identified in the gel stained with silver (data not shown). Proteins were extracted from each spot and subjected to mass spectrometry. One of the most consistently identified proteins was histone deacetylase 1 (HDAC1). To further confirm interaction of Fli1 with HDAC1 under physiological conditions, immunoprecipitation was performed using cell lysates prepared from quiescent dermal fibroblasts. As shown in Figure 1B, Fli1 formed a complex with HDAC1, but not with HDAC3. We next investigated if HDAC1 regulates acetylation levels of Fli1 by an *in vivo* acetylation assay using 293 T cells. Since PCAF mediates Fli1 acetylation, we examined the effect of forced expression of HDAC1 on acetylation levels of Fli1 in the presence of either PCAF or PCAF/ΔHAT. Expectedly, forced expression of HDAC1 markedly decreased the basal and PCAF-dependent acetylation of Fli1 (Figure 1C). To further confirm the involvement of HDAC1 in the regulation of Fli1 acetylation, we employed a gene-silencing technique using an antisense oligonucleotide. A pilot experiment established that the HDAC1 antisense oligonucleotide, which was previously shown to efficiently suppress the levels of HDAC1 protein [24], was superior in decreasing expression levels of HDAC1 in comparison with commercially available HDAC1 siRNA (data not shown). As shown in Figure 1D, the suppression of HDAC1 expression by the antisense oligonucleotide led to an increase of acetylation levels of Fli1, especially in the presence of PCAF. Taken together, these results indicate that HDAC1 interacts with Fli1 and subsequently mediates its deacetylation.

### p300 Promotes the Interaction of Fli1 with HDAC1 and Increases the DNA Binding Ability of Fli1 through Deacetylation of Lysine 380

**Figure 2:**
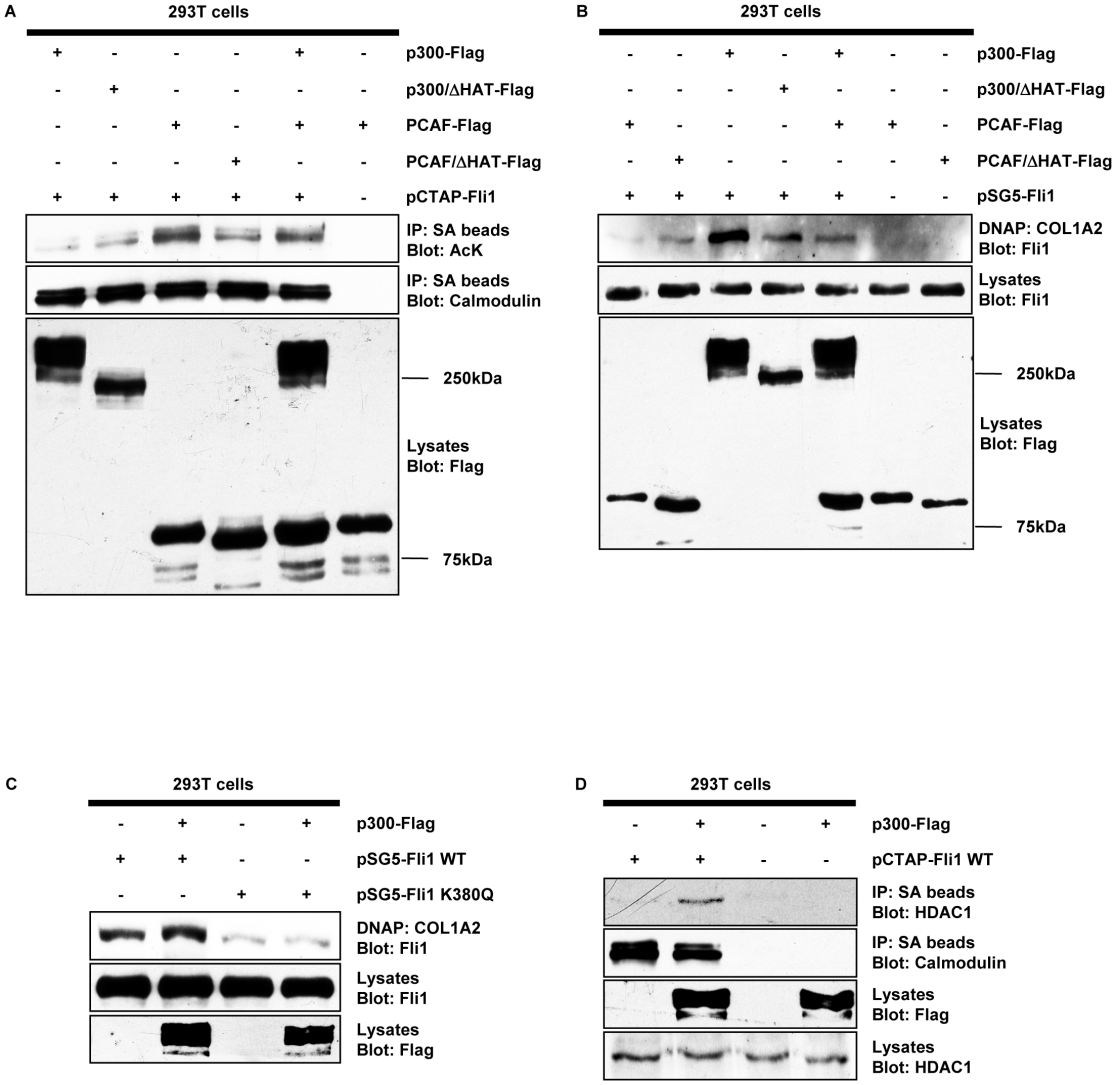
p300 increases the DNA binding ability of Fli1 through HDAC1-dependent deacetylation of lysine 380. A. 293T cells were transfected with pCTAP wild type Fli1 along with the indicated HAT proteins, and incubated for 48 hours. Total cell extracts were subjected to immunoprecipitation using streptavidin-coupled agarose beads (SA beads), followed by immunoblotting using rabbit anti-acetylated lysine antibody (AcK). In order to visualize the total levels of ectopically expressed Fli1, the same membrane was stripped and reprobed with anti-calmodulin binding peptide antibody. The levels of HAT proteins in cell lysates were determined by Western blotting. **B.** Whole cell lysates prepared as described above were incubated with biotin-labeled oligonucleotides. Proteins bound to these nucleotides were isolated with streptavidin-coupled agarose beads. The levels of Flag-tagged proteins and Fli1 in cell lysates were determined by Western blotting. **C.** 293T cells were transfected with pSG5 wild type Fli1 or Fli1 K380Q mutant along with the p300 expression vector, and incubated for 48 hours. **D.** 293T cells were transfected with pCTAP wild type Fli1 along with the p300 expression vector, and incubated for 48 hours. Total cell extracts were subjected to immunoprecipitation using SA beads, followed by immunoblotting using anti-HDAC1 antibody. To visualize the total levels of ectopically expressed Fli1, the same membrane was stripped and reprobed with anti-calmodulin binding peptide antibody. The levels of ectopically expressed p300 and endogenous HDAC1 in cell lysates were determined by Western blotting with anti-Flag antibody and anti-HDAC1 antibody, respectively.

Our previous studies have shown that PCAF-dependent acetylation of Fli1 at lysine 380 decreases its protein stability [20]. In the course of these experiments, we also noticed that p300 increases the protein stability of Fli1 (unpublished data). Given that acetylation of Fli1 decreases its protein stability [20], this observation suggested that p300 might influence Fli1 acetylation. To address this point, we carried out an *in vivo* acetylation assay using 293 T cells. As shown in Figure 2A, consistent with our previous report, forced expression of PCAF resulted in a robust increase of Fli1 acetylation. In contrast, low acetylation levels of Fli1 were observed in the presence of either p300 or p300/ΔHAT, suggesting that HAT activity of p300 does not acetylate Fli1. Importantly, PCAF-induced acetylation levels of Fli1 were decreased in the presence of p300, suggesting that p300 interferes with Fli1 acetylation by PCAF. Since acetylation reduces the DNA binding ability of Fli1 [20], we utilized DNAP to investigate the effect of p300 on the DNA binding ability of Fli1. As shown in Figure 2B, in contrast to PCAF, p300 increased DNA binding ability of Fli1. Furthermore, co-expression of p300 reversed the PCAF-dependent decrease of DNA binding of Fli1. To further investigate if p300 increases the DNA binding ability of Fli1 through deacetylation, we performed DNAP using a mutant of Fli1 (K380Q) that mimics the constitutively acetylated form of Fli1. As shown in Figure 2C, DNA binding of Fli1 K380Q mutant was markedly decreased compared with wild type Fli1, indicating that this mutant has the same property as the constitutively acetylated form of Fli1. In contrast to wild type Fli1, forced expression of p300 did not affect the DNA binding ability of Fli1 K380Q mutant, suggesting that p300 promotes the DNA binding of Fli1 by modulating acetylation status of Fli1. Given that p300 promoted deacetylation of Fli1 despite lacking histone deacetylase activity, it was speculated that p300 potentially enhances the interaction between Fli1 and HDAC1, leading to Fli1 deacetylation, or alternatively that p300 prevents Fli1 acetylation by competing for its interaction with PCAF. To address this point, we investigated the interaction of Fli1 with HDAC1 in the presence or absence of p300. Since 293T cells have a relatively low amount of HAT proteins, we employed this cell line for the experiment. As shown in Figure 2D, in the absence of p300, the interaction of Fli1 with HDAC1 was marginal. In contrast, forced expression of p300 resulted in increased interaction of Fli1 with HDAC1. On the other hand, in 293T cells transfected with Fli1 and PCAF, co-expression of p300 did not affect the interaction of Fli1 with PCAF (data not shown). Collectively, these results indicate that p300 promotes deacetylation of Fli1 by facilitating interaction of Fli1 with HDAC1, consistent with the increased DNA binding of Fli1.

### Phosphorylation of Fli1 at Threonine 312 Decreases its Interactions with p300 and HDAC1

**Figure 3:**
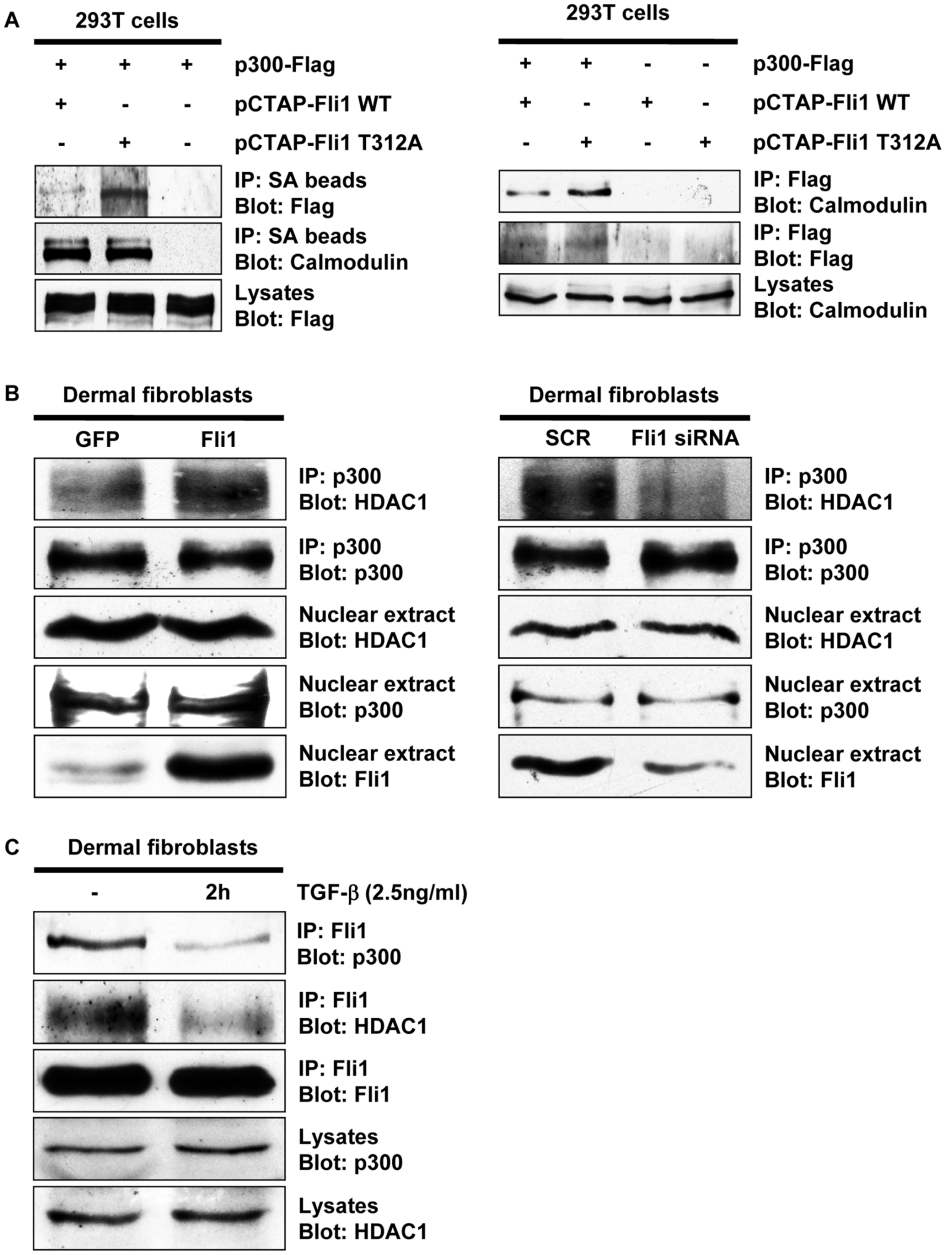
Phosphorylation of Fli1 at threonine 312 decreases its interactions with p300 and HDAC1. A. 293T cells were transfected with pCTAP wild type Fli1 or the Fli1 T312A mutant along with the p300 expression vector, and incubated for 48 hours. Total cell extracts were subjected to immunoprecipitation using streptavidin-coupled agarose beads (SA beads) followed by immunoblotting with anti-Flag antibody (left panels), or to immunoprecipitation using the anti-Flag antibody followed by immunoblotting with anti-calmodulin antibody (right panels). The levels of ectopically expressed p300 or Fli1 in cell lysates were determined by Western blotting with anti-Flag antibody (the bottom left panel) or anti-calmodulin antibody (the bottom right panel), respectively. **B.** Foreskin fibroblasts were transduced with 25 MOI of Fli1 or green fluorescence protein (GFP) adenovirus for 72 hours (left panels), or/and 25 MOI of Fli1siRNA or scrambled RNA (SCR) adenovirus for 72 hours (right panels). Nuclear extracts were subjected to immunoprecipitation with anti-p300 antibody, followed by immunoblotting with anti-HDAC1 antibody. The levels of Fli1, p300, and HDAC1 in nuclear extracts were evaluated by Western blotting. **C.** Whole cell lysates prepared from untreated or TGF-β stimulated foreskin fibroblasts were subjected to immunoprecipitation with anti-Fli1 antibody, followed by immunoblotting with antibodies against p300, HDAC1, or Fli1. The levels of endogenous p300 and HDAC1 were examined by Western blotting.

Previous studies have shown that phosphorylation of Fli1 at threonine 312 is required for its subsequent interaction with PCAF and acetylation at lysine 380 [21]. Since, as shown here, this process is also modulated by p300, we asked whether the Fli1 phosphorylation status affects its interaction with p300. To address this point, we compared complex formation of wild type Fli1 and a phosphorylation resistant mutant of Fli1 (Fli1 T312A) with p300 in 293T cells. As shown in Figure 3A, interaction with p300 was remarkably increased in the Fli1 T312A mutant compared with wild type Fli1, suggesting that the unphosphorylated form of Fli1 preferably forms a complex with p300. Next, we investigated whether Fli1 modulates the interaction of p300 with HDAC1. As shown in Figure 3B, p300 formed a complex with HDAC1. Importantly, forced expression of Fli1 increased this interaction (left panels), whereas gene silencing of Fli1 decreased the interaction of p300 with HDAC1 (right panels). Taken together, these results suggest that the unphosphorylated form of Fli1 recruits p300 and HDAC1 to form the transcription repressor complex. This notion was further confirmed under physiological conditions (Figure 3C). Since the peak of Fli1 phosphorylation occurs at 2 hours after TGF-β stimulation, we examined this time point. As predicted, association of Fli1 with p300 and HDAC1 was decreased at 2 hours after addition of TGF-β. Collectively, these results suggest that TGF-β-dependent phosphorylation of Fli1 at threonine 312 is a critical event regulating the remodeling of the Fli1 repressor complex.

### TGF-β-induced Histone H3 Acetylation Correlates with Fli1/HDAC1 Dissociation and Ets1/p300 Recruitment in the Context of the COL1A2 Promoter

**Figure 4:**
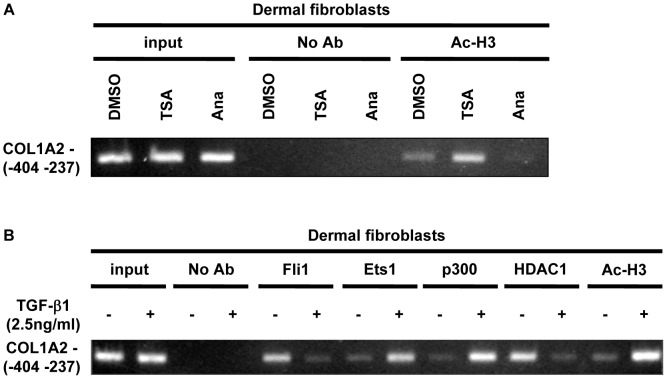
TGF-β-induced histone H3 acetylation correlates with Fli1/HDAC1 dissociation and Ets1/p300 recruitment to the COL1A2 promoter. A. Formaldehyde-cross-linked, moderately sheared chromatin was prepared from confluent quiescent fibroblasts treated with Trichostatin A (200 nM), anacardic acid (50 µM), or DMSO for 24 hours. The DNA fragments were immunoprecipitated using anti-acetylated histone H3 antibody, and the presence of the human α2(I) collagen (COL1A2) promoter fragments was detected using PCR. **B.** Formaldehyde-cross-linked, moderately sheared chromatin was prepared from foreskin fibroblasts untreated or treated with TGF-β1 (2.5 ng/ml) for 3 hours. The DNA fragments were immunoprecipitated using antibodies against Fli1, Ets1, p300, HDAC1, and acetylated histone H3. The presence of COL1A2 promoter fragments was detected using PCR.

Based on the current data described above, we hypothesized that Fli1 recruits HDAC1 to the COL1A2 promoter and promotes histone deacetylation and subsequent chromatin condensation, resulting in the suppression of COL1A2 gene expression. To assess this hypothesis, we first investigated the status of histone H3 acetylation around the Ets binding site in the COL1A2 promoter. As shown in Figure 4A, the acetylated form of histone H3 (Ac-H3) was detectable in this promoter region of unstimualted cells. Upon treatment with histone deacetylase inhibitor, Trichostatin A, the level of Ac-H3 increased, while after treatment with Anacardic acid, an inhibitor of HAT proteins, the level of Ac-H3 decreased, suggesting that this promoter region is subject to an active chromatin remodeling. Next, we examined the effect of TGF-β on the DNA occupancy of Fli1, Ets1, p300, and HDAC1 and on the level of Ac-H3 in the COL1A2 promoter. Consistent with our previous results, Fli1 occupied the COL1A2 promoter in unstimulated cells. Furthermore, in agreement with the *in vitro* findings HDAC1 was also present in this promoter region. However, there were only low levels of bound Ets1, p300, and Ac-H3 (Figure 4B). After 3 hours of TGF-β treatment DNA binding of Fli1 and HDAC1 was decreased, while DNA binding of Ets1 and p300 and the level of Ac-H3 were markedly increased. These results illustrate a dynamic remodeling of transcription factor complex on the COL1A2 promoter *in vivo* in response to TGF-β.

### HDAC1 Inhibits Collagen Gene Expression in Dermal Fibroblasts

**Figure 5:**
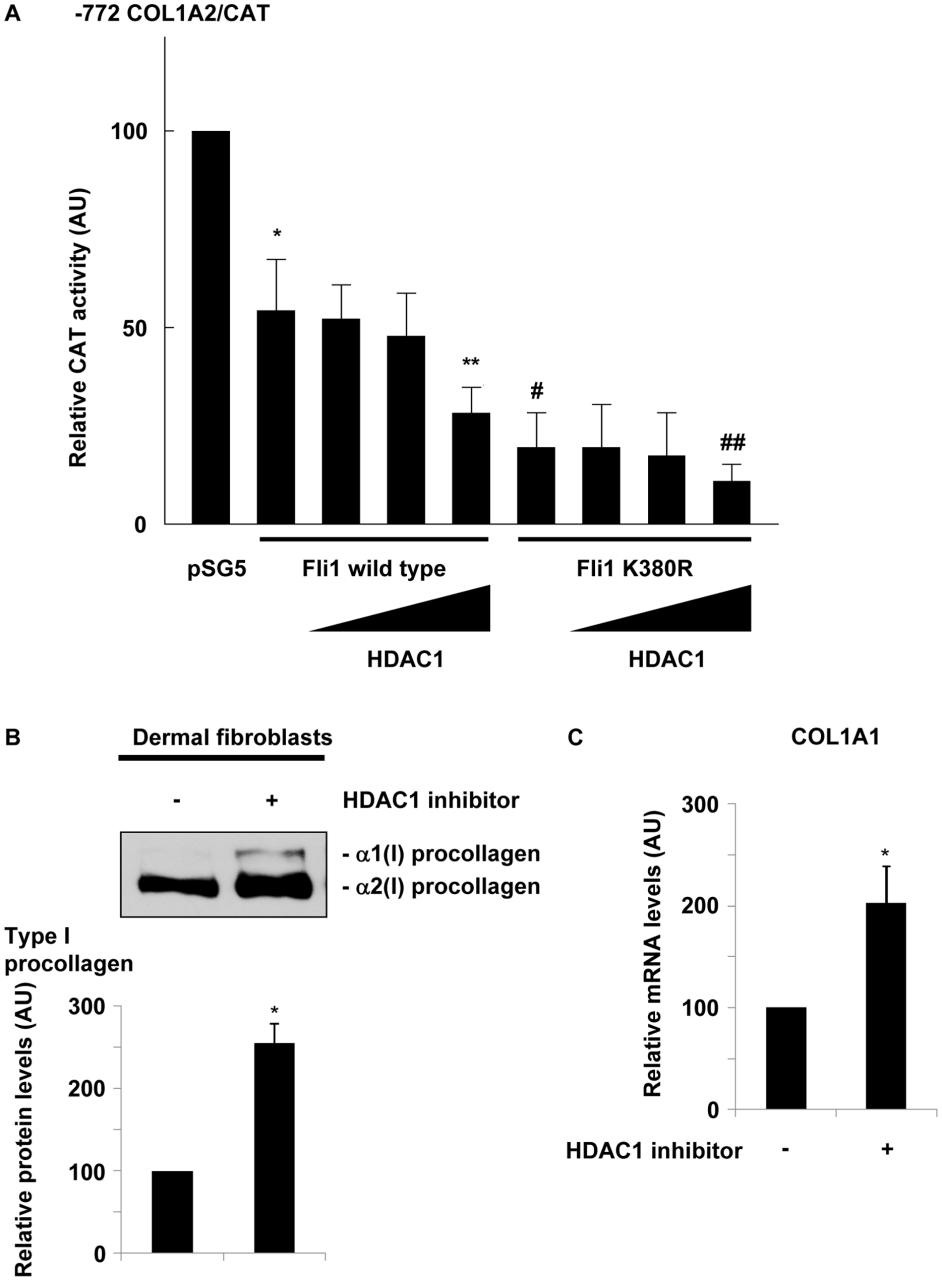
HDAC1 inhibits collagen gene expression in dermal fibroblasts. A. Foreskin fibroblasts were transfected with the −772 COL1A2/CAT construct (2 µg), along with wild type Fli1 or the Fli1 K380R mutant (0.1 µg) and HDAC1 (0, 0.1, 0.3, or 0.5 µg) for 48 h. Values represent CAT activities relative to those of untreated cells (100 arbitrary units [AU]). The mean and SD from three separate experiments are shown. * p<0.05 versus control cells. ** p<0.05 versus cells transfected with wild type Fli1 only. # p<0.05 versus control cells. ## p<0.05 versus cells transfected with Fli1 K380R only. **B.** Confluent quiescent foreskin fibroblasts were treated with HDAC1 inhibitor or vehicle for 24 hours. Type I procollagen protein levels in whole cell lysates were determined by immunoblotting. A representative result of three independent experiments is shown. The band density was evaluated by densitometry. **C.** Under the same conditions, mRNA levels of the α1(I) collagen (COL1A1) gene were determined using reverse transcription quantitative real-time PCR. The graph represents -fold change in COL1A1 mRNA levels in comparison to unstimulated controls, which were arbitrarily set at 100. The mean and SD from three separate experiments are shown. * p<0.05 versus control cells treated with vehicle.

To determine whether manipulation of HDAC1 has an effect on collagen gene expression, we investigated the impact of HDAC1 on the COL1A2 promoter activity. To this end, we employed a CAT reporter analysis using the −772 COL1A2/CAT construct. As shown in Figure 5A, overexpression of Fli1 suppressed the basal activity of the −772 COL1A2/CAT promoter. Expectedly, coexpression of HDAC1 with Fli1 further suppressed the promoter activity of the −772 COL1A2/CAT construct in a dose-dependent manner. These results suggest that HDAC1 magnifies the inhibitory effect of Fli1 on the promoter activity of the COL1A2 gene through an increase of DNA binding of Fli1 as well as histone deacetylation. To further confirm if chromatin remodeling is involved in the mechanism responsible for Fli1-dependent suppression of COL1A2 promoter activity, we used the Fli1 K380R mutant, in which lysine 380 is replaced with arginine. Since Fli1 is acetylated by PCAF at lysine 380, this construct is resistant to PCAF-dependent acetylation and its DNA binding ability to COL1A2 promoter is markedly increased. Consistent with our previous data [20], Fli1 K380R suppressed the basal promoter activity of the COL1A2 gene to a much greater extent than wild type Fli1. When HDAC1 was co-expressed, the inhibitory effect of Fli1 K380R was further magnified. Given that HDAC1 co-expression did not affect the DNA binding of the Fli1 K380R construct (data not shown), these results indicate that HDAC1 further strengthens the inhibitory effect of Fli1 on the promoter activity of COL1A2 gene through histone deacetylation.

To evaluate the effect of the inhibition of HDAC1 on type I collagen gene expression, we utilized the specific pharmacological inhibitor of HDAC1, entinostat. Human dermal fibroblasts were treated with 1 µM entinostat for 48 hours and type I collagen expression was examined by immunoblotting and reverse transcription quantitative real-time PCR. As shown in Figure 5B and 5C, blockade of HDAC1 by entinostat significantly increased mRNA and protein levels of the type I collagen gene. Collectively, these results indicate that HDAC1 largely contributes to the regulation of type I collagen gene expression through chromatin remodeling as a repressor complex “Fli1-p300-HDAC1” in human dermal fibroblasts.

## Discussion

**Figure 6:**
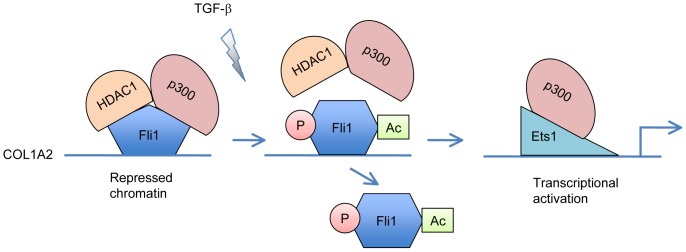
TGF-β-induced remodeling of the transcription factor complex at the Ets binding site in the human COL1A2 promoter. In quiescent cells, Fli1 forms a transcription repressor complex with p300 and HDAC1. HDAC1/p300 promotes deacetylation of Fli1, resulting in increased Fli1 DNA binding and a repressed chromatin state. After TGF-β stimulation, Fli1 phosphorylation by protein kinase C-δ induces disassembly of this transcription repressor complex and the acetylation of Fli1 by PCAF, leading to the loss of Fli1 DNA binding. Subsequently, the transcription activator complex consisting of Ets1 and p300 binds to the Ets binding site and activates transcription at least partially by promoting histone acetylation.

This study was undertaken to clarify the molecular mechanism by which Fli1 functions as a potent transcriptional repressor of the COL1A2 gene. We initially identified HDAC1 as a Fli1 interacting protein by combining tandem affinity purification and mass spectrometry. A series of experiments using an overexpression system in 293T cells demonstrated the following key findings: (i) the acetylation status of Fli1 is regulated by the balance between PCAF and HDAC1, (ii) p300 enhances the interaction between Fli1 and HDAC1 and Fli1 is maintained as a deacetylated form in the Fli1-HDAC1-p300 complex, resulting in an increase of its DNA binding ability, (iii) the unphosphorylated form of Fli1 preferably interacts with HDAC1 and p300. Given that TGF-β-dependent phosphorylation of Fli1 at threonine 312 promotes its interaction with PCAF and subsequently results in the loss of its DNA binding ability due to PCAF-dependent acetylation at lysine 380, it was speculated that TGF-β stimulation regulates the remodeling of the Fli1-HDAC1-p300 repressor complex through Fli1 phosphorylation at threonine 312. Supporting this idea, TGF-β decreased the interaction of Fli1 with HDAC1 and p300 in dermal fibroblasts at 2 hours after stimulation, when Fli1 phosphorylation at threonine 312 occurs. In ChIP analysis, more importantly, strong DNA binding of Fli1 and HDAC1 correlated with low levels of Ac-H3 in unstimualted dermal fibroblasts, while TGF-β-induced dissociation of Fli1 and HDAC1 and recruitment of Ets1 and p300 correlated with high levels of Ac-H3 (Figure 6). Given that forced expression of HDAC1 augmented the inhibitory effect of the Fli1 K380R mutant, whose DNA binding is not affected by HDAC1, on the COL1A2 promoter activity, Fli1 exerts its transcriptional repressor effect through HDAC1-dependent chromatin remodeling. Collectively, the current data clearly showed that Fli1 functions as a transcriptional repressor by promoting chromatin condensation due to HDAC1-dependent histone deacetylation and that phosphorylation of Fli1 at threonine 312 is a key post-translational modification regulating its association with HDAC1/p300 or with PCAF.

In this study, we observed converse binding of Fli1 and Ets1 to the COL1A2 promoter *in vivo*. This confirms previous *in vitro* studies that showed competition of Ets1 and Fli1 for the Ets binding site on the COL1A2 promoter [10]. While Ets1 has been shown to be a positive mediator of fibrosis [18], [25], its direct role in collagen gene regulation has not been fully defined, and surprisingly overexpression of Ets1 in dermal fibroblasts leads to inhibition of the COL1A2 gene [26]. Interestingly, recent ChIP-chip analysis of the Smad2/3 binding sites in HaCaT cells has revealed that the binding elements for Ets are significantly enriched in the Smad2/3 binding sites and knockdown of Ets1 results in overall alteration of TGF-β-induced transcription, suggesting that Ets contributes to the induction of the TGF-β-Smad pathways [27]. This global finding is consistent with earlier studies that showed cooperation of Ets1 and Smad3 on selected promoters, including the CCN2 gene [18]. Thus, it’s possible that in the context of the COL1A2 promoter Ets1 functions as a partner of Smad3 contributing to the recruitment of p300/CBP and chromatin remodeling.

The key role of p300/CBP as a positive regulator of collagen gene expression has been well documented [28]. Interestingly, our study suggests that p300 also functions as a facilitator of complex formation between Fli1 and HDAC1 and contributes to the Fli1-mediated transcriptional inhibition of the COL1A2 gene. While counterintuitive, this observation is consistent with previous finding that demonstrated interaction of the C/H3 domain of p300 with HDAC1 *in vitro* and *in vivo* [29]. Functionally, it was shown that the recruitment of HDAC1 to the C/H3 domain interferes with the process of autoacetylation and the function of p300 as transcriptional co-activator of MyoD and p53. A HAT/HDAC complex is also involved in FOXP3-mediated transcriptional repression. Specifically, FOXP3 recruits histone acetyltransferase TIP60 and HDAC7 to form a repressor complex to control development and function of regulatory T cells [30]. While in the present study we did not characterize the additional components of the Fli1/HDAC1/p300 complexes, it is likely to be a part of a multiprotein complex that includes additional chromatin remodeling factors. Interestingly, a study that focused on PCAF has demonstrated formation of large multiprotein complexes that include both HATs and HDACs and are distinct from the HDAC complexes containing mSin3A, Mi-2/NRD, or CoREST [31]. The mechanism of transcriptional regulation by the HAT/HDAC complexes is not yet fully understood, and further studies are needed to better understand the role of these complexes in the process of dynamic chromatin remodeling in response to different physiological and pathological stimuli [32].

HDAC inhibitors have shown promise as therapeutic agents in several diseases including cancer, heart hypertrophy, and organ fibrosis [33]. With respect to dermal fibrosis, it was shown that the class I/II HDAC inhibitor, Trichostatin A, suppresses TGF-β-induced collagen up-regulation in scleroderma fibroblasts by interfering with Smad signaling [34]. A follow up study has revealed that specific blockade of HDAC7 reproduced the anti-fibrotic effects of Trichostatin A, however the mechanism involved in inhibition of collagen genes by silencing HDAC7 has not been elucidated [35]. Our study demonstrates that specific inhibition of HDAC1 has a pro-fibrotic effect, also in scleroderma fibroblasts (data not shown). Since downregulation of Fli1 may contribute to scleroderma fibrosis [12], it may not be desirable to employ class I HDAC inhibitors as an anti-fibrotic treatment for scleroderma. However, further studies are needed to gain a more comprehensive understanding of the effects of specific HDAC inhibitors on fibroblast function in physiological and pathological conditions for potential future applications as effective anti-fibrotic treatments.

In summary, this is the first study reporting the detailed molecular mechanism by which Fli1 functions as a potent transcriptional repressor in the context of the COL1A2 promoter. This finding provides us a new clue to further understand the molecular mechanism underlying Fli1-dependent transcriptional regulation of various target genes.
